# Breast cancer occult in renal cell carcinoma: a case report of a rare tumor-to-tumor metastasis and literature review

**DOI:** 10.3389/fonc.2025.1710242

**Published:** 2025-12-16

**Authors:** Bin Huang, Yin Han, Yanjie Zhang, Hongsheng Liu

**Affiliations:** 1 Department of Pathology, The First People’s Hospital of Xiaoshan District, Hangzhou, Zhejiang, China; 2 Department of Pathology, Shanghai First Maternal and Infant Health Care Hospital, Shanghai, China; 3 Department of Pathology, The Second People’s Hospital of Xiaoshan District, Hangzhou, Zhejiang, China

**Keywords:** breast cancer, clear cell renal cell carcinoma, immunohistochemistry, metastasis, pathological diagnosis, tumor-to-tumor

## Abstract

Metastasis from breast cancer (BC) to renal cell carcinoma (RCC) is rare. We retrospectively analyzed the clinical data, i.e., medical history, imaging, and laboratory examinations, as well as pathological features of a 62-year-old female with invasive ductal carcinoma of the breast metastasizing to clear cell renal cell carcinoma (CCRCC). A comprehensive literature review was also conducted. Her right breast mass was pathologically diagnosed as invasive ductal carcinoma eight months ago. A large mass in the left kidney was incidentally discovered during a computed tomography (CT) scan, which was consistent with RCC. She underwent neoadjuvant chemotherapy, radical mastectomy, and endocrine therapy, as well as laparoscopic left nephrectomy four months postoperatively. Her pathological examination revealed two heterogeneous tumor components in the kidney: typical CCRCC [CD10+, Vimentin+, carbonic anhydrase IX (CAIX)+, paired box gene 8 (PAX-8)+, 3% Ki-67 proliferation index) as well as multifocal metastatic invasive ductal carcinoma of the breast (estrogen receptor (ER) 50%+++, GATA binding protein 3 (GATA3) +, Human epidermal growth factor receptor-2 (HER-2) 1+, and 20% Ki-67 proliferation index). These findings met the diagnostic criteria for TTM. She also continued BC endocrine therapy postoperatively. However, no recurrence or metastasis was observed during the 11 follow-up months.

## 1 Introduction

Breast cancer (BC) and renal cell carcinoma (RCC) are common clinical malignancies (1). Many studies have reported cases of multiple primary malignant tumors involving BC and RCC (2). However, in clinical practice, there is a distinct disease entity- tumor-to-tumor metastasis (TTM), which refers to the metastasis of one malignant tumor into another pre-existing tumor. However, very few studies have reported BC metastasizing to RCC.

In the TTM disease spectrum, RCC is the most common “recipient tumor”, and BC is one of the primary “donor tumors” (3, 4). A systematic literature review from 2000 to 2024 found only a few case reports (5–16). Analysis of these cases shows that among the donor tumors (BC), invasive ductal carcinoma reported the maximum number of cases, while invasive lobular carcinoma was reported in only one cases (13). Among the recipient tumors (RCC), clear cell renal cell carcinoma (CCRCC) was the main pathological subtype (10 cases), followed by one case of chromophobe cell carcinoma (9). Immunohistochemical results showed that ER, GATA3 and mammaglobin (MGB) were mostly expressed in metastatic BC, and CD10, Vimentin, CAIX and paired PAX-8 were commonly expressed in RCC. Additionally, a significant difference is observed in the Ki-67 proliferation index between the two (approximately <5% and ≥20% for CCRCC and primary BC), respectively (8, 10, 13, 15).

Since the disease lacks distinct clinical and imaging manifestations, it can be easily confused with double primary cancers and tumor heterogeneity. This can lead to missed diagnosis and misdiagnosis, thereby affecting treatment decisions. Therefore, we reported a case of invasive ductal carcinoma of the breast metastasizing to CCRCC, and analyzed its clinicopathological features, pathogenesis, and distinct diagnosis as well as treatment strategies in combination with the available literature. This might provide references for clinicians and pathologists for improving the diagnosis and prognosis of such patients.

## 2 Case presentation

Timeline showing the patient’s condition

- In 2014 [Disease discovery]: An asymptomatic mass was discovered in the right breast (not taken seriously and not treated).
- From January to February 2024 [Examination and diagnosis]: ① The right breast mass enlarged to 4.0×3.0×2.0 cm, and core needle biopsy confirmed the diagnosis of “invasive ductal carcinoma of the breast”, and ② CT incidentally detected a huge mass in the left kidney, which was consistent with RCC (not treated temporarily due to BC treatment).
- From February 2024 to May 2024 [Preoperative treatment]: she underwent six neoadjuvant chemotherapy cycles → the BC mass shrank to 2.5×1.5×1.0 cm.
- June 2024 [Surgery]: Post-modified radical mastectomy, the pathology confirmed invasive ductal carcinoma (WHO Grade 2). Endocrine therapy was performed according to the results of immunohistochemistry.
- October 2024 [Re-examination and evaluation]: She reported to the hospital for left kidney examination after more than three months postoperatively. ① Enhanced CT showed an 11.4×9.0×7.5 cm mass in the left kidney (consistent with CCRCC), and ② Positron emission tomography (PET)-CT excluded other systemic metastases.
- October 2024 [Surgery and diagnosis]: Post-laparoscopic left nephrectomy, the pathology confirmed kidney CCRCC+ Intrarenal metastatic BC, subsequently diagnosed as TTM.
- From October 2024 to September 2025 [Follow-up and treatment]: She continued BC endocrine therapy and was followed up for 11 months. No recurrence of perirenal/BC and distant metastasis was observed.

### General information

**Figure 1:**
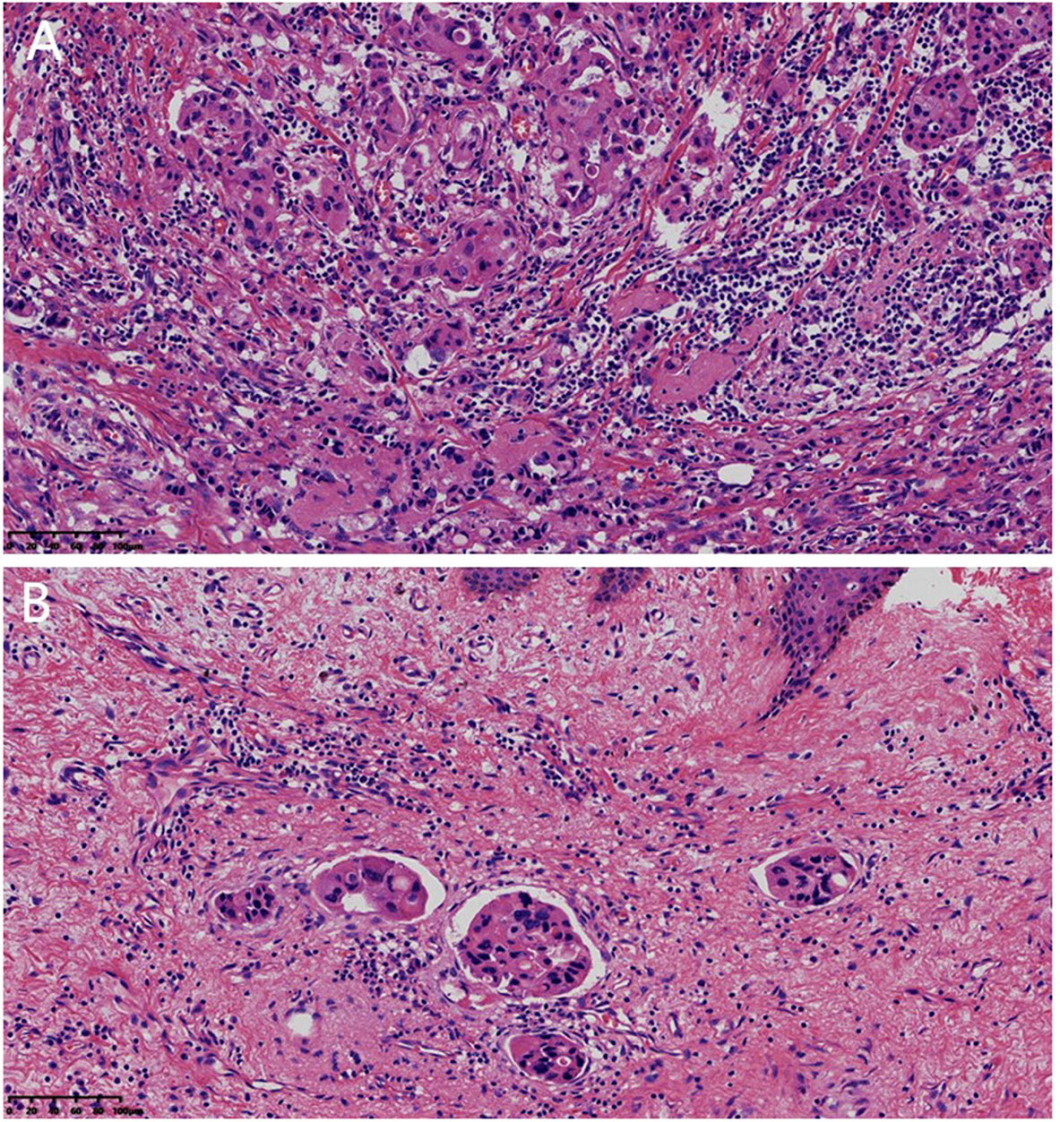
**(A)** Pathological section of modified radical mastectomy specimen for breast cancer: Changes of invasive ductal carcinoma after neoadjuvant chemotherapy (HE staining, ×200). **(B)** Lymphovascular tumor thrombi were present in the dermis (HE staining, ×200).

Our 62-year-old female patient noticed an asymptomatic mass in her right breast ten years ago, but did not undergo any medical evaluation. Eight months ago, due to the enlargement of the tumor to 4.0×3.0×2.0 cm, the histopathological evaluation of the biopsy specimen showed invasive ductal carcinoma. At that time, a CT examination incidentally found a huge mass in the left kidney, which was consistent with RCC (not treated temporarily due to BC treatment). The patient received six neoadjuvant chemotherapy cycles with the regimen: vinorelbine 40 mg/time + epirubicin 40 mg/time (intravenous drip, d1, d8, q3w). The tumor reduced to 2.5×1.5×1.0 cm in the size. After chemotherapy, she underwent modified radical mastectomy for BC in the hospital. Her postoperative pathology findings confirmed invasive ductal carcinoma (non-special type, WHO Grade 2) measuring 2.5×1.5×1 cm, with lymphovascular tumor thrombi in the dermis (**Figures 1A, B**); neoadjuvant treatment response graded as Miller-Payne Grade 3, and repeated axillary sampling revealed no lymph nodes. Her immunohistochemical results were as follows: ER (80%+++), PR (30%++), HER-2 (1+), GATA3 (+), and a 30% Ki-67 proliferation index. Additionally, fluorescence *in situ* hybridization (FISH) evaluation showed no HER-2 gene amplification (negative). Postoperatively, the patient was administered palbociclib capsules (125 mg once daily) and exemestane tablets (25 mg once daily) without any significant adverse reactions. More than 3 months after BC surgery, the patient’s condition remained stable, and she presented to our hospital. Her physical examination revealed flat and soft abdomen without tenderness or rebound tenderness, no percussion pain in the bilateral renal regions, postsurgical scarring of the right breast without any palpable mass, and no enlarged superficial lymph nodes were detected in the body. Past medical history comprised hypertension for over ten years (managed with enalapril and valsartan, dosages unspecified) and type 2 diabetes mellitus for more than a year (not treated). No family history of genetic diseases or cancer was reported.

### 2.2 Imaging examinations

**Figure 2:**
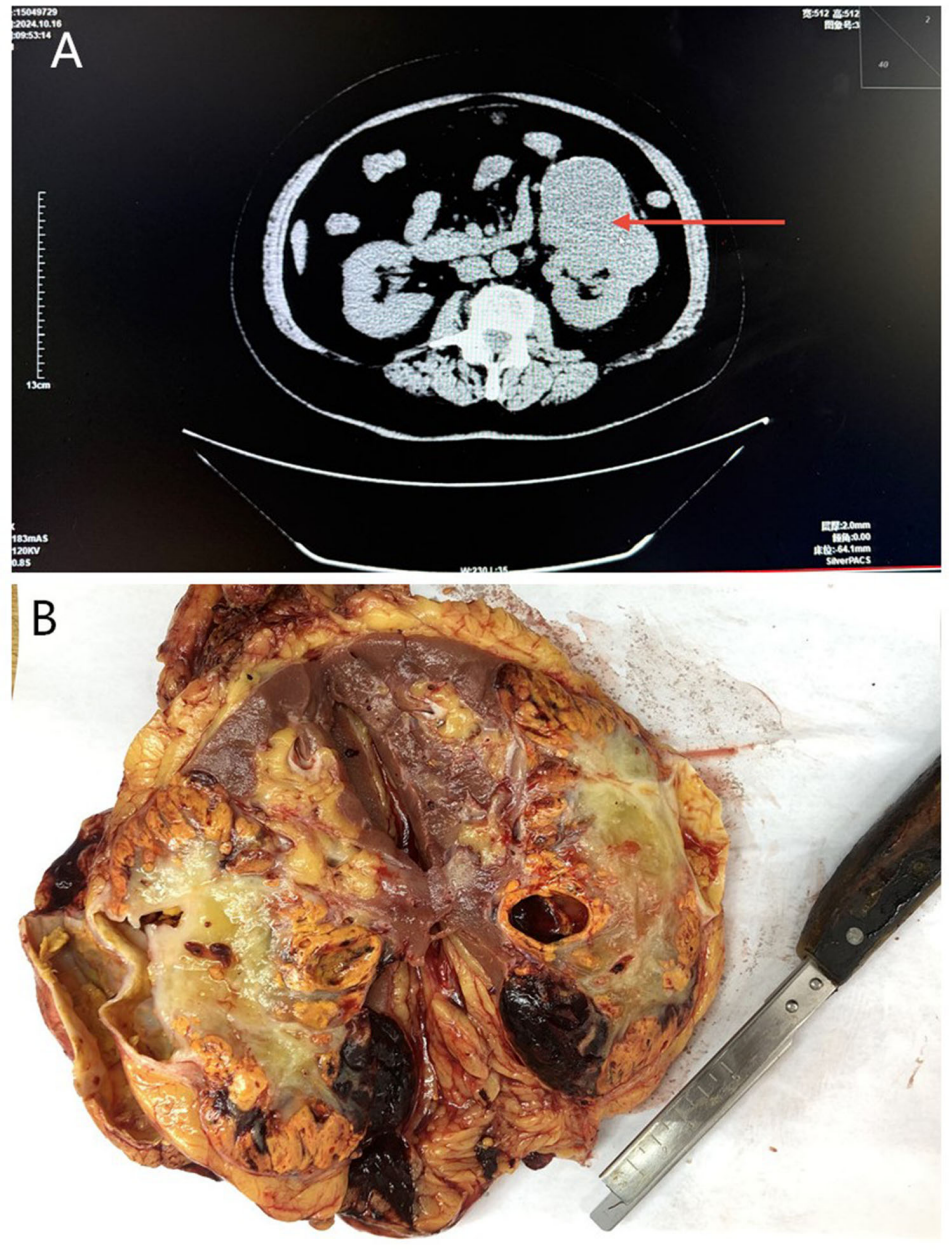
**(A)** Enhanced CT of the urinary system: An irregular mass measuring 11.4×9.0×7.5 cm was observed in the left renal parenchyma (red arrow), accompanied by cystic changes and calcification. The right kidney appeared normal. **(B)** Gross examination of the left renal tumor: The cut surface showed golden-yellow and grayish-white colors, with hemorrhage and multilocular cysts, and ill-defined boundaries.

Enhanced CT of the urinary system (**Figure 2A**) revealed an irregular mass-like lesion with abnormal density (11.4×9.0×7.5 cm) in the left renal parenchyma, accompanied by cystic changes and calcification. Having ill-defined borders, the lesion was consistent with CCRCC. However, the right kidney showed normal size and density.

In the PET-CT scan, no significant increase in fluorodeoxyglucose (FDG) uptake was detected in the postoperative right breast region. Although the left renal lesion was consistent with CCRCC with cystic changes, no malignant findings were identified in other body parts.

### 2.3 Laboratory investigations

Her routine urine tests, comprising urine glucose, occult blood, and protein, were negative. However, serum tumor markers like CEA, CA199, AFP, squamous cell carcinoma-related antigen) were within normal limits.

### 2.4 Surgery and pathological examination

A laparoscopic left nephrectomy was performed under general anesthesia. During the operation, the left kidney and tumor were completely resected, and no renal hilar lymph nodes were not touched.

Pathological tissues were fixed with 10% neutral formalin for 24 h at 25 °C, embedded in paraffin, and 3 μm continuous sections were prepared for hematoxylin-eosin staining (HE) (Shanghai Rego Biotechnology Development Co., Ltd., and Sinopharm Chemical Reagent Co., Ltd.). The tissues were observed using a Leica DM2000 light microscope (Leica Microsystems GmbH).

The gross examination (**Figure 2B**) revealed that the kidney’s volume was 14.0×9.0×8.5 cm. An 11.5×9.0×7.0 cm mass was found in the middle and lower poles of the renal parenchyma. Although the cut surface was golden-yellow and grayish-white, it displayed dark red hemorrhagic areas, with ill-defined boundaries and soft texture. It exhibited numerous cysts of varying sizes (0.5-6.0 cm in diameter). Some contained light red fluid, and no obvious invasion of the renal pelvis and calyces was observed. The ureter and the renal blood vessel were 5.0 cm (0.6 cm in diameter) and 4.0 cm (0.2 cm in diameter) long, respectively. However, no enlarged hilar and perirenal lymph nodes were detected.

**Figure 3:**
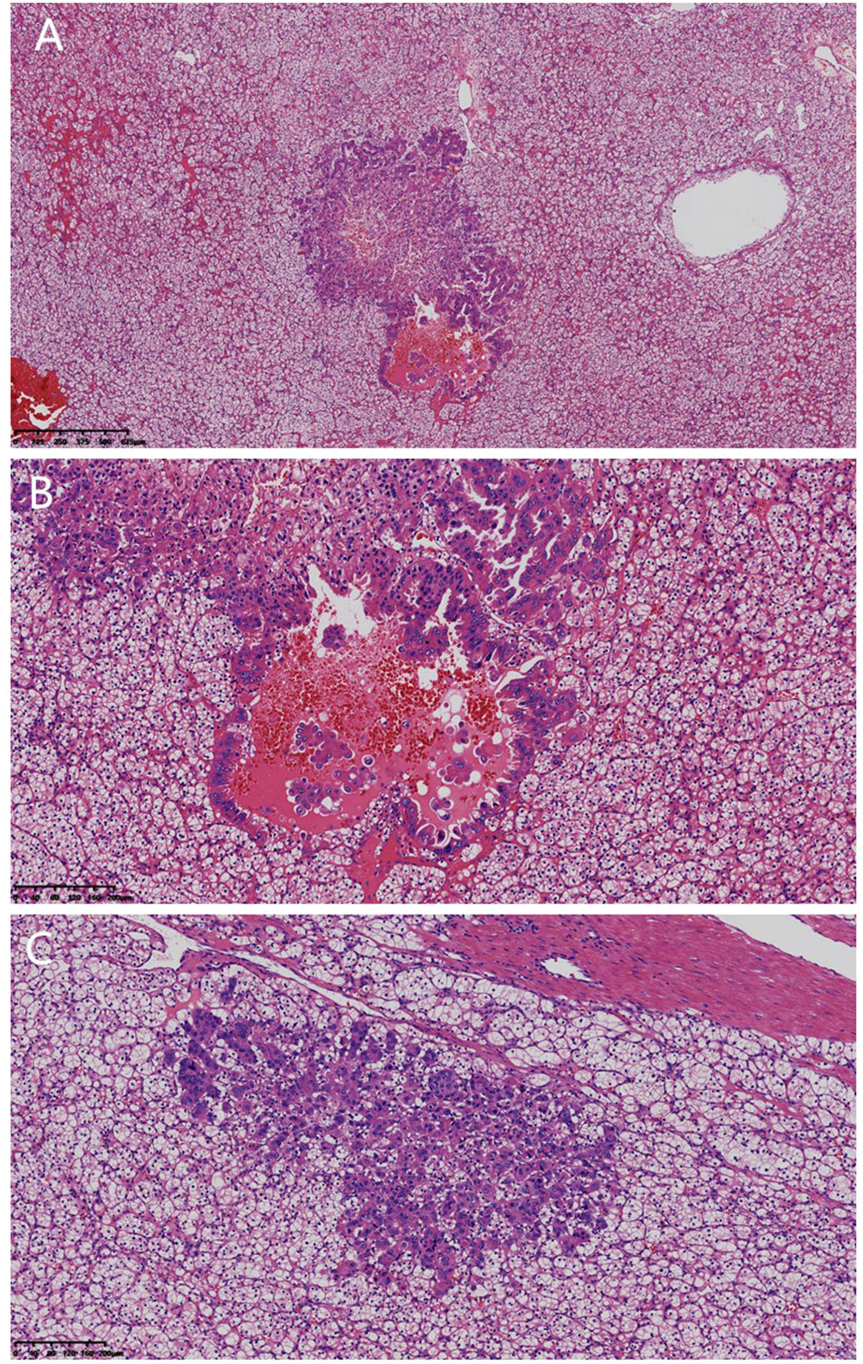
**(A)** Microscopic view of the tumor showing two distinct components: The surrounding area consisted of CCRCC growing in solid sheets, while the central area was a metastatic focus of breast cancer (HE staining, ×32). **(B)** Metastatic focus: It exhibited glandular-tubular and micropapillary structures, with moderately pleomorphic cells and distinct nucleoli (HE staining, ×100). **(C)** The central area was a metastatic focus of breast cancer, surrounded by CCRCC (HE staining, ×100).

Microscopic examination (**Figures 3A–C**): The tumor displayed two heterogeneous components: ① The main component was typical CCRCC, which displayed a solid sheet shape, with clear cytoplasm, slight atypia, and unobvious nucleoli (WHO/ISUP Grade 1); ② Multifocal metastatic lesions in CCRCC, which showed small nest-like, glandular-tubular, micropapillary, and irregular cystic structures, with local hemorrhage, moderate cell atypia, abundant cytoplasm, few large nuclei, clear nucleoli, and increased nuclear-cytoplasmic ratio. However, no lymphovascular tumor thrombi or necrosis was observed in either component.

**Figure 4:**
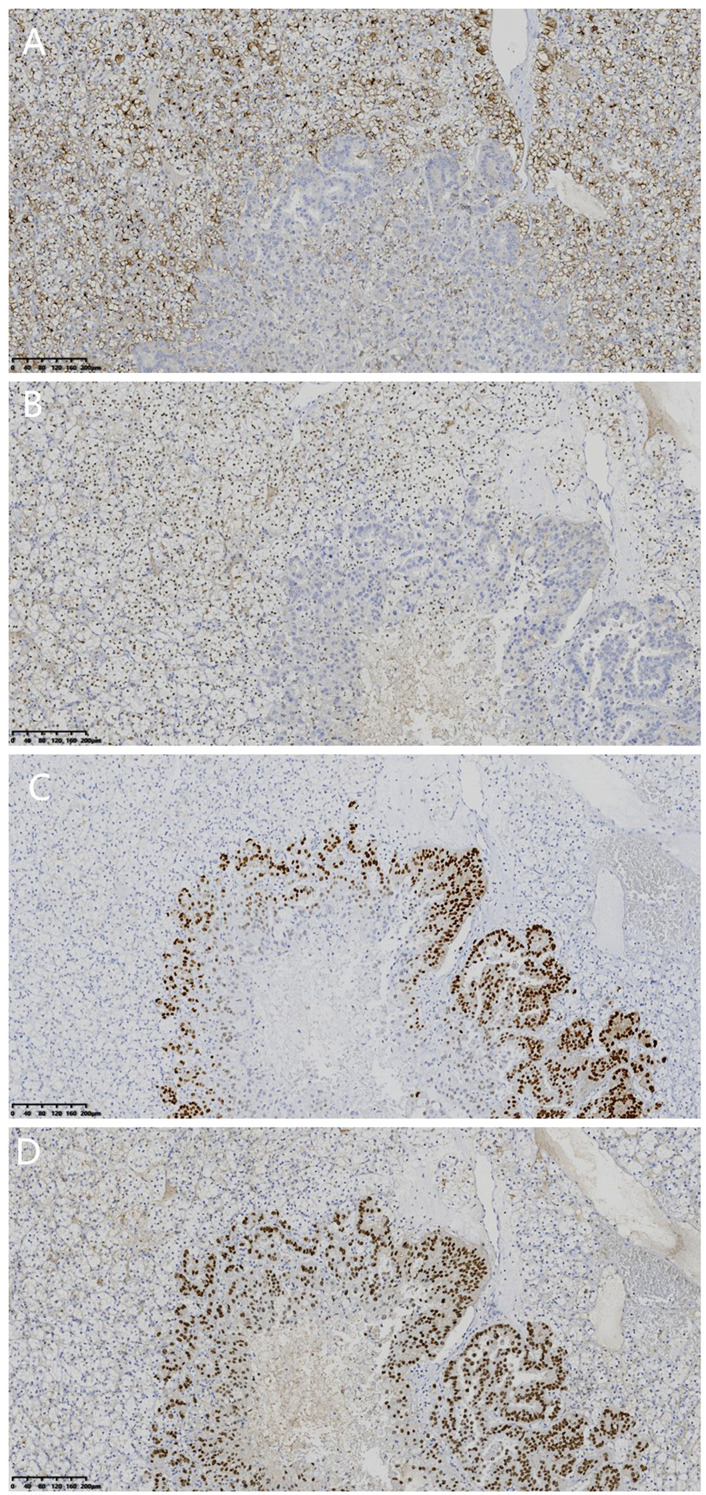
**(A)** Immunohistochemistry showed diffuse positive staining for CD10 in CCRCC, whereas CD10 staining was negative in metastatic lesions. (×200). **(B)** Immunohistochemistry showed nuclear staining of PAX-8 in CCRCC, while no PAX-8 staining was observed in metastatic lesions. (×200). **(C)** Immunohistochemistry showed strong nuclear staining of ER in 50% of metastatic lesions, whereas ER staining was negative in CCRCC. (×200). **(D)** Immunohistochemistry showed nuclear expression of GATA3 in the metastatic lesion, whereas GATA3 staining was negative in CCRCC. (×200).

Immunohistochemical results are as follows (**Table 1**, **Figures 4A–D**):

**Table 1:** Immunohistochemical findings of the tumor components in this case.

Marker	ccRcc component	Breast cancer metastasis to ccRcc internal components	Breast cancer
CD10	+	–	/
Vimentin	+	–	/
CAIX	+	–	/
PAX-8	+	–	/
p504S	–	–	/
CD117	–	–	/
ER	–	50%+++	80%+++
PR	–	–	30%++
GATA3	–	+	+
HER2	–	1+	1+
p120	–	CM +	CM +
EMA	+	+	+
E-cadherin	+	+	+
Ki-67	3%	20%	30%

CM, Cell membrane; /: No done.

CCRCC component showed CD10 (+), Vimentin (+), CAIX (+), PAX-8 (+), p504S (-), CD117 (-), and 3% Ki-67 proliferation index while metastatic BC component exhibited ER (50%+++), GATA3 (+), PR (-), HER-2 (1+), p120 (cell membrane +), and 20% Ki-67 proliferation index.

Both components showed EMA (+) and E-cadherin (+) expressions.

Her pathological diagnosis was: CCRCC of the left kidney (pT2bN0M0, WHO/ISUP Grade 1), with multifocal metastatic invasive ductal carcinoma of the breast, and BC stage was revised to pT2N0M1.

### 2.5 Treatment and follow-up

The patient did not receive any distinct treatment after nephrectomy and followed the post-BC surgery treatment regimen (oral palbociclib + exemestane). By September 2025 (11 months post-nephrectomy), regular re-examinations showed no recurrence of perirenal and BC as well as no distant metastasis.

## 3 Discussion

According to the 2022 global cancer statistics, BC and RCC ranked second and 14th among all malignant tumors, accounting for 11.6% and 2.2% of all malignancies, respectively (1). Clinically, these two tumors have generated multiple primary malignancies (2), but as a special entity, “tumor-tumor metastasis (TTM)”. The TTM phenomenon entails metastasis of one malignant tumor into another existing tumor; however, it is very rare. RCC is the most common “receptor tumor”, accounting for 38.8%. Moreover, BC is the most crucial “donor tumor”, accounting for 12.7% of all malignancies, much higher than other tumor types (3, 4). Of them, BC, being a “donor tumor”, can metastasize to a subtype of RCC as a “recipient tumor”. According to the relevant literature from 2000 to 2024, only a few cases have been reported globally (5–16). Therefore, this report has focused on a case of invasive ductal carcinoma of the breast with metastasis to CCRCC in combination with previous literature. We have also systematically analyzed the diagnostic criteria, clinicopathological features, pathogenesis, and clinical management strategies of this type of TTM to provide a valuable reference for clinicians and pathologists in diagnosis and treatment.

### 3.1 Diagnostic basis of TTM in this patient

TTM must be diagnosed using the classic criteria proposed by Campbell et al. (17)and updated later (18): The donor and recipient tumors are both independent primary malignant tumors; Metastasis secondary to a primary lymphatic system malignancy is excluded; There is clear evidence of the donor tumor’s primary focus; The two tumors’ morphology and immunophenotype are consistent, and the metastatic focus is partially surrounded by the recipient tumor tissue.

Our case met the above criteria: There was no histological association between the donor tumor, which was invasive ductal carcinoma of the right breast (with clear surgical pathological evidence in 2024), and the recipient tumor, which was left kidney CCRCC (diagnosed by surgical pathology in 2024); No history of malignant tumor of the lymphatic system; The pathological features of the primary focus of BC (ER/PR positive, GATA3+) were highly consistent with the immunophenotype of the kidney’s metastatic focus (ER 50%+++, GATA3+), and The metastatic focus was multifocal in CCRCC and surrounded by clear cell components, thereby excluding the possibility of a collision tumor or continuous invasion.

### 3.2 Clinicopathological features of BC metastasizing to RCC

The fundamental pathological diagnosis for BC metastasizing to RCC lies in the “recognition of dual components plus immunohistochemical verification”. Combined with the 12 previously reported cases and this case, a total of 13 TTM cases of BC metastasis to RCC were identified. Their core features were as follows (**Table 2**). Patient characteristics: All patients were female, with ages ranging from 43 to 79 years. Their median and average age were 62 and 62.5 years, respectively. Although renal tumors were predominantly asymptomatic, only one case exhibited painless hematuria (8). Most of the renal space-occupying lesions were detected via imaging examinations during BC follow-up; their imaging manifestations showed no significant differences from other types of renal cancer. Notably, one case was incidentally discovered during autopsy (5), while another one was unexpectedly found in a pulmonary metastatic focus of CCRCC (15).

**Table 2:** Summary of cases of breast cancer metastasis to renal-cell carcinoma.

Year/no.	Author	Age/sex	Types of breast cancer	Types of RCC	Site of metastasis	Transfer interval (years)	Treatment	Follow-up(M)
2001/4.	Val-Bernal JF ,et al. (5)	75/F	IDC	CCRCC	LR	30	Autopsy findings	NM
2004/5.	Van Wynsberge LK ,et al. (6)	64/F	IDC	CCRCC	LR	6	R	NM
2006/6.	Möller MG ,et al. (7)	62/F	IDC	CCRCC	RR	2	R	10 D
2008/7.	Ulamec M ,et al. (8)	60/F	IDC with neuroendocrine differentiation	CCRCC	LR	0(S)	R+ M + RT+ET	18 NR
2011/8.	Perrin C ,et al. (9)	49/F	IDC	CCRCC	RR	0(S)	R	NM
2015/9.	Huo Z ,et al. (10)	43/F	IDC	CCRCC	LR	4	PR+C+ET	3 Disease progression
2016/10.	M Urdiales-Viedma ,et al. (11)	71/F	IDC	CCRCC	LR	12	R	NM
2017/11.	W. Toro-Zambrano et al. (12)	54/F	IDC	chromophobe cc	RR	0(S)	R	NM
2019/12.	Lakovschek I-C ,et al. (13)	79/F	Bilateral IDC	CCRCC	RR	0(S)	R+ M + TT+ ET+RT	36 D
2020/13.	Ashman D ,et al. (14)	57/F	ILC	CCRCC	RR	11	ET	Stable disease
2021/14.	Lima Á ,et al. (15)	57/F	IDC	CCRCC	Left lung (within ccRCC metastasis)	1	RPM +RT+ET+ Anti-Her2+ TT	NM
2024/15.	S. Geetha,et al. (16)	79/F	BC	RCC	RR	NM	NM	NM
2025	present	62/F	IDC	CCRCC	LR	<1	R+ ET	11 NR

BC, Breast Cancer; IDC, Invasive ductal carcinoma; ILC, Invasive lobular carcinoma; CCRCC, Clear cell renal cell carcinoma; L, Left; R, Right; S, Synchronization; PN, Partial Nephrectomy; CT, chemotherapy; RT, Radiation therapy; M, Mastectomy; ET, Endocrine Therapy; Targeted TT, Therapy; RPM, Resection of pulmonary metastases; NM, Not mentioned; D, Die; NR, Not Recurrence.

Tumor pathological subtypes: In BC subtypes, invasive ductal carcinoma was the predominant type (11/12), including one case with neuroendocrine differentiation (8), followed by invasive lobular carcinoma (1/12) (14).

RCC subtypes: Among all RCC cases, 11 were CCRCC(11/12), and one was chromophobe cell carcinoma (1/12) (12).

Immunophenotypic features:

Metastatic BC components: These components frequently expressed breast-specific markers, including ER, PR, GATA 3, and MGB. The positive rates of ER and PR were 83.3% (10/12) and 61.5% (5/9), respectively. However, only two cases were positive for HER-2 (5, 12). These findings were partially consistent with our case (ER+, PR-, HER-2 1+).

RCC recipient components: These components expressed RCC-specific markers, like CD10, Vimentin, CAIX, and PAX-8.

Ki-67 proliferation index: The two components showed a significant difference in the Ki-67 proliferation index. The Ki-67 proliferation index of RCC was usually <5%, while that of primary BC was mostly ≥20% (8, 10, 13, 15). This was consistent with our case, where the Ki-67 proliferation index was 3% and 30% for RCC and primary BC, respectively. Metastatic interval: Synchronous metastasis, i.e., simultaneous diagnosis of BC and RCC, was observed in three cases. Metachronous metastasis (diagnostic interval of 1–30 years between the two cancers) was observed in eight cases. Since the interval in our case was eight months, it was classified as short-interval metastasis.

### 3.3 Pathogenesis of TTM

Currently, the TTM mechanisms are primarily based on the “seed-and-soil” theory (19) and the “mechanical theory”. These theories are elaborated below: The “seed-and-soil” theory: This theory proposes that donor tumor cells (the “seeds”) should adapt to the recipient tumor’s microenvironment (the “soil”) to complete metastatic colonization. In the scenario where CCRCC serves as the recipient tumor, due to the inactivation of the von Hippel-Lindau (VHL) gene in CCRCC, hypoxia-inducible factor (HIF) is overexpressed abnormally. This promotes the massive secretion of vascular endothelial growth factor (VEGF) and helps in creating a highly vascularized microenvironment (20). Since CCRCC cells are rich in glycogen and lipids, they can provide energy for metastatic cancer cells. Collectively, these factors create a “fertile soil” for metastatic colonization.

In our case, the primary BC focus was associated with lymphovascular tumor thrombi and a Ki-67 proliferation index of 30%, indicating that the “seeds” (BC cells) displayed strong metastatic potential. The “seed” characteristics matched the “soil” features of CCRCC, such as low-grade malignancy (WHO/ISUP grade 1) and a Ki-67 proliferation index of 3%, thereby facilitating metastasis.

The mechanical theory: The kidneys receive approximately 20% of the cardiac output (21). Additionally, the highly vascularized nature of CCRCC increases the probability of capturing circulating tumor cells (CTCs). In the present case, renal metastasis was detected eight months after the diagnosis of BC, which may be related to the colonization of CTCs in CCRCC through the systemic circulation.

### 3.4 Clinical management and prognosis

The treatment strategy of BC metastasis to RCC should consider the “dual tumor characteristics”. RCC is primarily treated by surgical resection (especially localized lesions), while BC is mainly treated by surgery, followed by endocrine therapy, targeted therapy, or chemoradiotherapy, based on molecular typing (6–15).

The presence of systemic metastasis and the sensitivity of BC to treatment are crucial for prognosis. Six patients were followed up for 3 months to 36 months. One patient had stable disease, two had no recurrence (including this case),one had disease progression, and two died (7, 8, 10, 13, 14).

This case report offers notable clinical value for addressing diagnostic and therapeutic challenges of the rare TTM between BC and RCC. Clinically, this TTM is easily misdiagnosed as double primary cancers or tumor heterogeneity due to nonspecific features. By detailing the integrated diagnostic process—combining medical history, imaging, pathological identification of dual tumor components, and immunohistochemical verification (e.g., ER/GATA3 for metastatic BC, CD10/CAIX/PAX-8 for clear cell RCC, and distinct Ki-67 indices)—it provides a practical paradigm for clinicians and pathologists. For BC patients with renal space-occupying lesions, it highlights the need to consider TTM in differential diagnosis and prioritize surgical pathological confirmation to avoid misdiagnosis or improper treatment. Additionally, its analysis of BC subtype-tailored endocrine therapy and 11-month disease-free survival offers actionable guidance for clinical management. Given the scarcity of reported cases, this work enriches understanding of TTM’s clinicopathological features, pathogenesis, and prognosis, serving as a valuable reference for improving diagnosis accuracy and treatment efficacy of similar rare cases.

## 4 Conclusion

BC metastasizing to RCC is an extremely rare TTM phenomenon and can be easily missed clinically. Hence, its diagnosis requires amalgamation of medical history, imaging findings, pathological examination of both components, and immunohistochemistry. TTM should be considered in patients with a history of BC and report renal space-occupying lesions. Thus, surgical resection of the renal lesion should be prioritized to confirm the diagnosis. Subsequent treatment plans should be tailored to the molecular BC subtype; a long-term follow-up can improve the patient’s prognosis. This case provides clinical references for diagnosing and treating such rare diseases; however, multi-center studies are crucial to explore the biological behavior and optimal treatment strategies of this condition because of the limited sample size.

## Data Availability

The raw data supporting the conclusions of this article will be made available by the authors, without undue reservation.
